# The Fluorescence Detection of Phenolic Compounds in *Plicosepalus curviflorus* Extract Using Biosynthesized ZnO Nanoparticles and Their Biomedical Potential

**DOI:** 10.3390/plants11030361

**Published:** 2022-01-28

**Authors:** Musarat Amina, Nawal M. Al Musayeib, Nawal A. Alarfaj, Maha F. El-Tohamy, Gadah A. Al-Hamoud, Muneerah K. M. Alqenaei

**Affiliations:** 1 Department of Pharmacognosy, Pharmacy College, King Saud University, Riyadh 11451, Saudi Arabia; galhamoud@ksu.edu.sa; 2 Department of Chemistry, College of Science, King Saud University, Riyadh 11451, Saudi Arabia; nalarfaj@ksu.edu.sa (N.A.A.); moraby@ksu.edu.sa (M.F.E.-T.); 3 Department of Botany and Microbiology, College of Science, King Saud University, Riyadh 11451, Saudi Arabia; muneerahalqenaie@gmail.com

**Keywords:** *Plicosepalus curviflorus*, ZnO nanoparticles, fluorescence, antibacterial, anticancer, hemolysis activity

## Abstract

A facile, eco-friendly fluorescence approach based on the biogenic formation of zinc oxide nanoparticles using the biomass of *Plicosepalus curviflorus* shoots was developed. The suggested approach was employed to analyze three phenolic compounds (catechin, curviflorside, and curviflorin) isolated from the shoots of *P. curviflorus*. The surface morphology of the prepared ZnONPs was characterized by carrying out different microscopic and spectroscopic investigations. A significant UV-Vis absorption peak of ZnONPs was recognized at 345 nm and the FT-IR spectra of the isolated catechin, curviflorside, and curviflorin in the presence of sodium dodecyl sulfate (SDS) and ZnONPs were recorded at λem 470, 490, and 484 nm after excitation at λex 380, 420, and 410 nm. The suggested fluorescence method displayed linear concentration ranges of 10–120, 5–100, and 10–150 μg mL^−1^ for the three isolated compounds, respectively. The shoot extract, isolated compounds, and ZnONPs were screened for antibacterial and anticancer effects against four different types of bacterial strains and HeLa cells, respectively. The ZnONPs exhibited the highest zone of inhibition against *Escherichia coli* and *Staphylococcus aureus* strains when compared with pure, isolated compounds and shoot extract. The anticancer potential of ZnONPs (64%) was stronger as compared to the 160 µg mL^−1^ of shoot extract (49%), catechin (52%), curviflorside (54%), and curviflorin (58%) at 160 µg mL^−1^. Moreover, all the samples were investigated for hemolysis activity and showed a potent anti-hemolytic effect. The developed analytical method showed excellent sensitivity and reliability for the concurrent analysis of the isolated bioactive markers.

## 1. Introduction

Nowadays, enormous focus on the progress of powerful, inexpensive, and sensitive identification techniques for analyzing natural isolated chemical makers, preferably bioactive ones in herbal extracts for their acceptance in global markets [1,2]. Several advanced analytical techniques, including chromatographic (HPLC, UPLC, HPTLC) separations [3], electrochemical analysis [4], surface plasmon resonance [5], quartz crystal microbalance [6], and optical methods such as chemiluminescence and fluorescence are used for the bioactive markers detection and quantification based on the principle of different corresponding signal appearance [7,8]. The fluorescence detection is notably the most executed optical method for the analysis of bioactive components due to its superiorselectivity and sensitivity [9,10]. Despite, successful development in fluorescence techniques in the various bioactive components detection, certain limitations in signal amplification and unfavorable noisy backgrounds still exist [11]. Hence, there is an urgent need to design ultrasensitive strategies to improve detection performance. Various strategies have been employed to solve these problems, including the synthesis of new fluorescent substances, the improvement of instruments for fluorescence detection, and the establishment of an integrated analysis system [12,13,14]. Recently, the use of functionalized nanomaterials in fluorescence detection techniques has provided a new opportunity for the selective and ultrasensitive detection and quantification of biomarkers. The advancement in the making and designing of nanostructures has resulted in the rapid development of several nanoparticles with unique chemical and physical features such as a large surface area and distinctive optical, magnetic, and electronic properties [15]. These nanoparticles have a unique biocompatible nature that makes them highly suitable for bio-detection applications. Several nanostructures such as carbon nanomaterials [16], noble metal and metal oxide nanoparticles [17], and nanocrystal semiconductor [18] are utilized successfully in the biomarker detection by fluorescence detection probes. Nanomaterials such as metal and metal oxides have enhanced the sensitivity and selectivity of detection because of their huge surface area/volume ratio, high interaction, size-dependent characteristics, and a high degree of functionalization [19]. In addition to this, organic ligand attachment, covalent functionalization, and the formation of nanocomposites have made huge contributions to upgrading the response sensitivity and selectivity in biomolecules detection [20]. The optical and electronic features of the nanostructures involved in fluorescence detection have greatly improved their reproducibility and limit of detection for the selection quantification of biomolecules [21]. Recently, zinc oxide nanoparticles with unique inherent properties such as easy synthesis, facile functionalization, outstanding fluorescence intensity, rapid dissolution, the quick formation of ZnO ions, biocompatibility, and stability have grabbed enormous interest in the areas of energy storage, photocatalysis, optical devices, and signal detection [22,23]. ZnO nanostructures in quantum size have served as fluorescent labels for a pancreatic cancer marker detection, carbohydrate antigen 19-9 [24]. N-Doped ZnO nanoparticles based on optical biosynthesis have been used for the selective detection of urea [25]. Furthermore, studies in the literature have claimed that ZnONPs prepared from plant extracts have elevated the fluorescence detection as well as the biological properties of the ZnONPs due to the presence of fluorescent active phytocompounds in the plant extracts [26,27,28].

Several studies on fluorescence enhancement systems triggered by ZnONPs have been reported in the literature [29,30] but their application to the detection of natural compounds in herbal extracts or formulations is still unknown. In the present study, we were encouraged to develop an advanced fluorescence detection probe for the ultrasensitive quantification of natural isolates in *P. curviflorus* extract using ZnONPs.

*Plicosepalus curviflorus* (Benth. ex Oliv.) Tiegh., a semi-parasitic plant and member of the Loranthaceae family, is a source of interesting bioactive constituents with notable medicinal features [31,32,33]. *P. curviflorus* naturally grows in Saudi Arabia, Yemen, East Africa, and Northeast Africa [34]. The entire plant has been utilized in traditional medicine to cure diabetes [35] and improve lactation in cattle [34] in Saudi Arabia. In Yemen, the stem and heated twigs of this plant have been used in cancer treatment and as a poultice on the chest to treat pneumonia, respectively [36]. The flavane gallates, triterpenes, quercetin, catechin, and sterols present in the leaf and stem extracts possess antioxidant, hypoglycemic, antibacterial, cytotoxic, and notable anti-diabetic properties [37,38,39]. Moreover, green-synthesized ZnONPs and natural plant-derived triterpenes (catechin) have enormous potential in various biological aspects, including antimicrobial, anticancer, immunomodulatory, biological labeling, biological sensing, drug delivery, gene delivery, and nanomedicines [40,41,42,43].

The promising chemotaxonomic and medicinal value of *P. curviflorus*, as well as the great potential of ZnONPs to provide fluorescence signals with high emission tenability and tremendous medicinal value, has encouraged us to utilize this plant for the preparation of zinc oxide nanoparticles. The objective of the present study is to exploit a new and simple ultrasensitive eco-friendly fluorescence method based on ZnONPs, with specific emission features and desirable size to detect three natural phenolic compounds isolated from the shoots of *P. curviflorus* and phytochemically confirmed by NMR spectra. The biosynthesized ZnO nanoparticles, ethyl acetate extract, and isolated compounds were screened for antibacterial, anticancer, and hemolytic activities.

## 2. Materials and Methods

### 2.1. Apparatus

A Perkin-Elmer (Model LC-34, Waltham, MA, USA) polarimeter was used to determine the specific optical rotations. Fourier transform infrared analysis was measured using a Perkin-Elmer (PerkinElmer Ltd., Yokohama, Japan) spectrophotometer. IR spectra were obtained on a 400-Infrared (IR) Shimadzu spectrometer (Shimadzu, Kyoto, Japan) and ultraviolet spectra were obtained in methanol using Lambda 25UV/VIS, Perkin-Elmer spectrometer (Waltham, MA, USA). ESI-MS analyses were measured on a triple, TSQ-7000 quadruple stage-MAT mass spectrometer (Thermo, Berman, Germany), and HR-ESI-MS patterns were recorded on Orbitrap-LTQ mass spectrometer (Thermo Fisher, Waltham, Massachusetts, United States). The Bruker DRX 700 spectrometer (Bruker, Reinstten, Germany) was used to measure NMR (1H and 13C) spectra in dimethyl sulfoxide-d6 and deuterated chloroform solvents. The 2D NMR spectra (HSQC, HMBC, COSY, and NOESY) were measured on the same instrument. Merck SiO_2_ gel of mesh 0.04-0.063 mm size (Darmstadt, Germany) and RP18 SiO_2_ gel (75 µm particle size, Merck, Darmstadt, Germany) were employed for chromatographic separations. Pre-coated 60-F254 SiO_2_ gel Merck and F_254_ RP-18 plates (Merck, Darmstadt, Germany) were spotted for the thin layer chromatographic (TLC) analysis. Thin-layer chromatography zones were visualized by derivatizing sheets with a p-anisaldehyde/sulfuric acid reagent and spot development appeared at 110 °C. The spectroscopic characterization of ZnONPs was performed on a 2100-Biochrom Ultrospec (Biochrom Ltd., Cambium, Cambridge, United Kingdom) spectrophotometer for UV-Vis spectrum detection. The pattern of X-ray diffraction (XRD) was recorded by a D-5000 Siemens (Siemens, Erfurt, Germany) diffractometer. X-ray photoelectron spectroscopy (XPS) was recorded by Ultra Axis Kratos X-ray spectroscopy (Kratos Analytical Ltd., Manchester, UK). The Raman spectrum was obtained by using a micro-Raman spectrometer (CRAIC Technologies, CA, USA). Energy-dispersive X-ray spectroscopy (EDX) was performed on 7610F-JSM (JEOL, Tokyo, Japan). The size distribution and zeta potential (ZP) of the synthesized ZnONPs were calculated by dynamic light scattering (DLS) on Malvern Zetasizer NanoZS Nano series (Worcestershire, UK). However, the size, shape, and surface distribution of the synthesized ZnONPs were determined by a transmission electron microscope (TEM), JEOL-1200EX model instrument (JEOL Ltd., Freising, Germany), and scanning electron microscope (SEM), model JSM-7610F (JEOL, CA, USA), to confirm the synthesis of the nanoparticles.

### 2.2. Chemicals and Reagents

Methanol (96.0%), n-hexane (95.0%), chloroform (96.0%), ethyl acetate (99.0%), n-Butanol (95.5%), acetone (99.0%), acetone-d6 (99.5%), methanol-d4 (99.8%), dimethyl sulfoxide (DMSO, 99.9%), p-anisaldehyde (98.0%), sulfuric acid (95.0%), zinc acetate dihydrate (Zn(CH3COO)2.2H2O) (99.9%), sodium dodecyl sulfate (SDS), 3-(4,5-dimethylthiazol-2-yl)-2,5-diphenyl-2H-tetrazolium bromide (MTT), 2% osmium tetroxide, 2% triton x-100, 3% glutaraldehyde, Dulbecco’s Modified Eagle Medium (DMEM), and 10% of fetal bovine serum (FBS) were purchased from Sigma-Aldrich (Hamburg, Germany). Tryptone Soya Agar (TSA) was purchased from Himedia (Mumbai, India), Brain Heart Infusion broth (BHI) was obtained from Oxoid (Oxoid Ltd., Basingstoke, UK), Mueller–Hinton Agar was acquired from Kasvi, Italy. Ciprofloxacin was purchased from local drug stores.

### 2.3. Botanical Material

The shoots of *P. curviflorus* (500 g) were collected in March 2018 from Abha, Saudi Arabia. Professor Dr. Mohamed Yousef at the Pharmacognosy Department, Faculty of Pharmacy, KSU, taxonomically identified the botanical material. Voucher specimen number PC-3-2010, describing the plant, is deposited in the same department.

### 2.4. Microbial Pathogens and Cancer Cell Line

Four bacterial strains, including *Staphylococcus aureus* (*S. aureus*, *ATCC 29213*), *Escherichia coli* (*E. coli*, *ATCC 25922*), *Enterococcus faecalis* (*E. faecalis*, *ATCC 29212*), and *Pseudomonas aeruginosa* (*P. aeruginosa*, *ATCC 27853*), were supplied by the Microbiology Department of King Khalid Hospital, Riyadh, Saudi Arabia. The HeLa (human cervical cancer cells) cancer cells were procured from the King Khalid Hospital Research Center (KKHRC), Riyadh, Saudi Arabia.

### 2.5. Extraction and Isolation Procedures

The methanol extract was obtained by the maceration of dried powder shoots of *P. curviflorus* (500 g) in methanol (2 × 3 L, each) using a soxhlet apparatus at ambient temperature for 8 h. The selection of solvent was based on solubility and safety, and solvents with polarity values near to the solute polarity perform better extraction. Alcohols (MeOH and EtOH) are considered universal solvents for the extraction of phytoconstituent investigations. The resulting methanol extract was filtered and then freed from the organic solvent by evaporation under reduced pressure at 50 °C on the rotator evaporator, affording a dark green 14.0 g of methanol extract. This crude methanol extract was dissolved in distilled H_2_O (150 mL) and consequently fractionated with (3 × 400 mL) n-hexane, (3 × 400 mL) ethyl acetate, and (3 × 400 mL) n-butanol to obtain 4.1 g of n-hexane, 2.9 g of ethyl acetate, 2.6 g of n-BuOH, and 4.6 g of aqueous fractions, respectively, after the removal of the solvents in a vacuum. The EtOAc soluble fraction was taken up for vacuum liquid chromatography using the stepwise gradient CHCl_3_: MeOH (9:1→1:1) to obtain five subtraction PC1-PC5. The PC2 subtraction (232 g) was loaded on a SiO_2_ gel (50 g × 50 × 2 cm) column using gradient n-hexane: ethyl acetate to afford compound **1** (10.9 mg) as an amorphous yellow solid. PC2 subtraction (345 mg) was treated in a similar way and compound **2** (34.7 mg) was obtained as a yellow amorphous solid. Subtraction PC3 (541 mg) was loaded on a Sephadex LH-20 column. MeOH was used as an eluting solvent to obtain two main fractions, PC-3A (261 mg) and PC-3B (196 mg). Furthermore, subtraction PC-3A was chromatographed on the RP18 column (50 g × 2 cm) eluting with a MeOH: H_2_O gradient to yield compound **3** (4.6 mg) as an amorphous white powder. The purification of PC-3B was carried out in a similar way as PC-3A, resulting in the isolation of compound **4** (6.2 mg) as an amorphous white powder.

### 2.6. Spectral Analysis of Isolated Compounds

**Figure 1:**
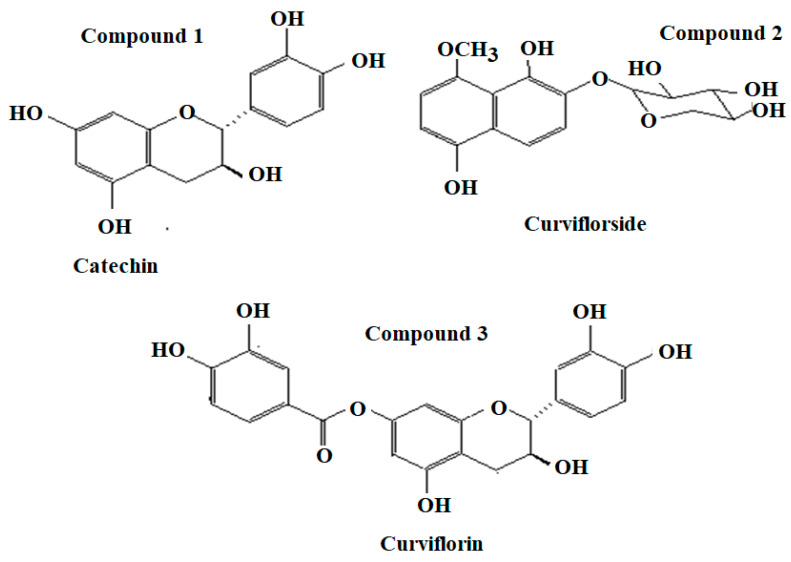
Chemical structures of Compound **1**: Catechin; compound **2**: Curviflorside; and compound **3**: Curviflorin.

Catechin 1: Amorphous yellow powder; [α]D: -20.2 (c 0.04, acetone); MS/ESI: *m*/*z* 301 [M + Na]^+^, 290, 255 [M + H]^+^ (calcd for C15H14O6); IR (KBr) νmax: 3400, 1622, 1523, 1460, 1240, 1130, 1060, 830 cm^−1^; UV (MeOH) λmax (log ε): 220 (4.25), 290 (2.65) nm; 1H NMR data (700 MHz, acetone-d6) δH (ppm): 2.46 (1H, dd, H-4α), 2.89 (4H, dd, H-β), 3.98 (1H, ddd, H-3), 4.56 (1H, d, H-2), 5.94 (1H, s, H-6), 5.82 (1H, s, H-8); 13C NMR (700 MHz, acetone-d6) δH (ppm): 28.83 (C-4), 67.92 (C-3), 80.96 (C-2), 94.84 (C-6), 95.98 (C-8), 101.45 (C-10), 115.12 (C-2′), 117.26 (C-5′), 122.02 (C-6′), 130.32(C-1′), 145.05 (C-4′),145.43 (C-3′), 153.65 (C-9), 156.04 (C-5), 156.12(C-7). Based on NMR data and comparison with available literature compound **1** was identified as catechin [39] (Figure 1).

Curviflorside 2: Yellow amorphous powder; [α]D: +52.3 (c 0.06, MeOH); HR-ESI-MS: *m*/*z* 339.1075 (Calcd. for C16H19O8 [M + H]^+^, 339.1080); IR (KBr) νmax: 3425, 2934, 1625,1518, 1070 cm^−1^; UV (MeOH) λmax (log ε): 237 (5.36), 258 (4.04), 297 (3.78) nm; 1H NMR data (700 MHz, DMSO-d6) δH (ppm): 7.55 (1H, d, *J* = 8.2 Hz, H-3), 7.70 (1H, d, *J* = 8.2 Hz, H-4), 7.47 (1H, d, *J* = 8.5 Hz, H-6,7), 5.00 (1H, *J* = 7.5 Hz, H-1′), 3.41 (1H, m, H-2′), 3.25 (1H, m, H-3′), 3.43 (1H, m, H-4′), 3.86 (1H, m, H-5′), 4.05 (3H, 8-OCH3), 13C NMR (175 MHz, DMSO-d6) δC (ppm): 152.6 (C-1), 140.1(C-2), 112.0 (C-3), 112.8 (C-4), 149.0 (C-5), 110.7 (C-6), 109.1 (C-7), 148.9 (C-8), 108.1 (C-9), 123.5 (C-10), 103.3 (C-1′), 73.5 (C-2′), 76.0 (C-3′), 69.8 (C-4′), 66.1 (C-5′), 61.4 (OCH3). Based on spectral data, the compound was identified as 1,5-dihydroxy-8-methoxynaphthalene-2-O-β-D-xylopyranoside [38] (Figure 1).

Curviflorin 3: Amorphous white solid; [α]D: +15.3 (c 0.5, MeOH); HR-ESI-MS: *m*/*z* 427.1033 (calcd for C22H19O9, [M + H]^+^, 427.1029); IR (KBr) νmax: 3356, 1732, 1614, 1519, 1463 cm^−1^; UV (MeOH) λmax (log ε): 218 (4.86), 277 (2.96) nm; 1H NMR data (700 MHz, DMSO-d6) δH (ppm): 4.64 (1H, d, *J* = 7.2 Hz, H-2), 3.94 (1H, m, H-3), 2.67 (1H, dd, *J* = 5.2 Hz, Hα-4), 2.45 (1H, dd, *J* = 8.2 Hz, Hβ-4), 6.13 (1H, d, *J* = 2.0, H-6), 6.21 (1H, d, *J* = 2.0, H-6), 6.75 (1H, brs, H-1′), 6.68 (1H, d, *J* = 7.8, H-5′), 6.58 (1H, d, *J* = 7.8, H-6′), 7.09 (1H, d, *J* = 2.2, H-2″), 6.95 (1H, d, J = 8.2, H-5″), 7.06 (1H, dd, *J* = 8.2, 2.2, H-6), 5.75 (brs, 3-OH), 9.75 (s, 5-OH), 8.87 (s, 3″-OH), 9.38 (s, 4″-OH); 13C NMR (175 MHz, DMSO-d6) δC (ppm): 81.6 (C-2), 66.4 (C-3), 28.1 (C-4), 155.6 (C-5), 100.6 (C-6), 150.7 (C-7), 101.6 (C-8), 156.6 (C-9), 106.2 (C-10), 130.7 (C-1′), 114.9 (C-2′),145.4 (C-3′), 146.1(C-4′), 115.6 (C-5′), 118.9 (C-6′), 139.7(C-1″), 109.5 (C-2″), 145.9 (C-3″), 146.1(C-4″), 108.0 (C-5″), 109.5 (C-6″), 164.9(C-7″). Based on spectral data the compound was identified as (+)-catechin-7- O-3″,4″-dihydroxybenzoate [38] (Figure 1). 2.7. Preparation of P. curviflorus Biomass

After harvesting, dried shoots of *P. curviflorus* were collected, thoroughly washed with distilled water, and then kept for drying in an oven for one week at 40 °C. The dried biomass was coarsely powdered to gain a uniform powder. The obtained powder was soaked in 95% methanol for 48 h. The gained *P. curviflorus* biomass was then utilized for the production of ZnONPs.

### 2.7. Green Preparation of ZnONPs Using Biomass of P. curviflorus

**Scheme 1:**
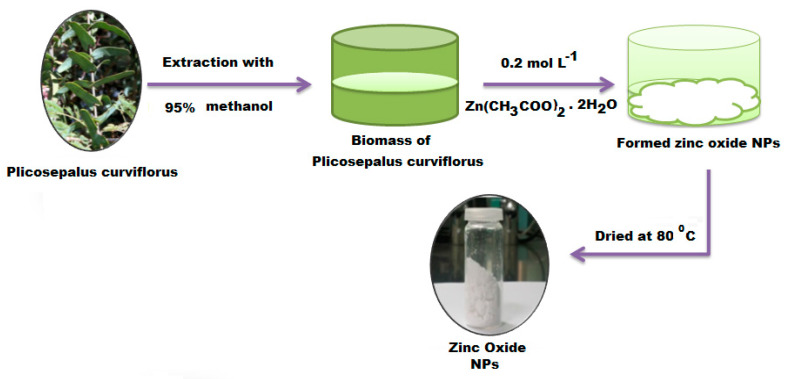
Biosynthesis steps for the green synthesis of ZnONPs using *P. curviflorus biomass*.

The eco-friendly synthesis of ZnONPs was conducted by adding 0.2 mol L^−1^ of zinc acetate dihydrate in methanol at room temperature and ultrasonically at 25 °C for 2 h. The above precursor solution was added to 100 mL of *P. curviflorus* shoots methanol biomass. The mixture was kept under continuous stirring for 20 min to obtain the ZnO nanoparticles precipitate. The obtained precipitate was centrifuged and washed with an excess of methanol several times, followed by deionized water, and then oven dried for 4 h at 80 °C (Scheme 1).

### 2.8. Spectroscopic and Microscopic Characterization of ZnONPs

The formation of ZnONPs was confirmed by UV-Vis spectroscopy in a 200 to 800 nm absorption wavelength. FTIR analysis in 400–4000 cm^−1^ range was applied to detect the functional moieties of the *P. curviflorus* biomass used for the synthesis of ZnONPs. The stability and the size distribution by the intensity of the prepared ZnONPs were assessed by zeta potential (ZP) and dynamic light scattering (DLS). Cu Kα radiation was applied for XRD analysis to detect the crystalline structure of ZnONPs at 40 kV and 25 mA. The morphological shape and particle size distribution of the green-designed ZnONPs were characterized by SEM equipped with EDX. The shape and size of the ZnONPs were also examined under TEM at different magnifications.

### 2.9. Fluorescence Analysis

The fluorescence intensity (FI), for the determination of catechin, curviflorside, and curviflorin in the presence of surface-active agent sodium dodecyl sulfate and ZnONPs, was tested in optimum fluorescence conditions at room temperature. To quantify the investigated constituents in their original plant extract, aliquots of the working solutions in the ranges of 10–120, 5–100, and 10–150 µg mL^−1^ were transferred into 10-mL volumetric flasks and phosphate buffer was used to adjust the pH of each solution to be 6. The FI was recorded at λ_ex_ 380, 420, and 410 and λ_em_ 470, 490, and 484 nm for catechin, curviflorside, and curviflorin, respectively.

### 2.10. Constructed Calibration Graphs

Under optimal conditions, the construction of calibration curves for catechin, curviflorside, and curviflorin was plotted using three different concentrations of the investigated catechin, curviflorside, and curviflorin solutions vs. FI at 12 experimental points. Each testing sample measurement was performed in triplicate. The graphs were fitted using least square linear regression.

### 2.11. Antibacterial Activity

Disk diffusion and agar well diffusion methods were applied to evaluate the bacterial susceptibility and to choose the test sample concentrations for the broth dilution procedure. The antibacterial activity was tested against four different clinically important bacterial species, *S. aureus*, *E. faecalis*, *E. coli*, and *P. aeruginosa*. The selection was based on the more suscitable (E. faecalis and E. coli) and more resistant (S. aureus and P. aeruginosa) strains in each of the Gram-positive and Gram-negative groups of bacteria. The bacterial strains were cultured on Tryptone Soya Agar and incubated for 24 h at 35 °C. The cultures were stored in a refrigerator at 4 °C until the working cultures were prepared. The working solution cultures were prepared by mixing a small quantity of culture stock with Brain Heart Infusion broth (BHI, 5 mL) and incubated for 24 h at 35 °C. The disc diffusion assay was performed by uniform spreading the working cultures onto the agar plates surface. An amount of 10 µL of each test sample suspension was impregnated onto the inoculated agar surface by using 9 mm diameter sterile discs of filter paper. In the case of the well diffusion method, the working cultures were placed onto the surface of agar plates. After the solidification of agar, a 5 mm diameter well was prepared aseptically and filled with a 32 µL sterile suspension of each test sample. Mueller–Hinton Agar was used for both the assays. A concentration of 10^9^ CFU mL^−1^ was chosen as an initial cell concentration and three different concentrations (30, 60, and 120 µg mL^−1^) were selected as sample concentrations. Ciprofloxacin (0.02 mg mL^−1^) and Millipore water were used as positive and negative controls, respectively. After 48 h incubation at 35 °C, the zone of inhibition around the discs and wells was noticed.

### 2.12. Determination of Baterostatic and Bacteriocidal Concentration

The minimum inhibitory concentration (MIC) and minimum bactericidal concentration (MBC) of the test samples against *S. aureus* and *P. aeruginosa* were conducted by a microdilution broth assay using Mueller–Hinton broth at pH 7.2. The initial concentration of sample (4096 µg mL^−1^) was prepared in sterile water and serially diluted in a 96-plate by twofold using Muller-Hinton broth. The final concentration was obtained between 2048 to 1.0 µg mL^−1^ range. During the investigation, ciprofloxacin was applied as positive control. 0.5 McFarland suspension with 1–2 × 10^8^ CFU/mL density in Mueller–Hinton broth was used to prepare a standard by direct colony suspension. A 5.0 × 10^5^ CFU/mL as final test concentration was obtained by diluting the prepared suspension in each well, followed by 18 h incubation at 35°C under moist condition. The minimum concentration of pathogen to inhibit the complete visible growth of bacteria in the well plate was used to calculate the MIC value, while the Minimum Bactericidal Concentration (MBC) was measured as the lowest concentration at which no bacterial colonies were detected in the after 10^−1^ dilution and 18 h incubation.

### 2.13. Morphological Investigation of P. aeruginosa and S. aureus

The change in morphology of treated S. aureus and P. aeruginosa with plant extract and ZnONPs in contrast with untreated S. aureus and P. aeruginosa strains was investigated under SEM. 5–10 mm pieces of each treated bacterial strains were prepared and maintained in phosphate-buffered saline solution (pH 7.4) containing 3% glutaraldehyde. Afterwards, 2% osmium tetroxide was used for another 1 h fixation. Finally, ethanol was applied to dehydrate the treated tissues, dried in a humidified incubator at ambient temperature with CO_2_ flow and viewed under SEM at 15 kV ac-celeration voltage. 

### 2.14. Hemolytic Activity

Hemolysis tests were carried out using human red blood cells (RBCs) at a total concentration of 6.62 × 10^12^ RBCsL^−1^ and total hemoglobin of 199 gL^−1^, 0.01 × 10^9^ WBCsL^−1^ to examine the hemolytic activity of the ethyl acetate extract, isolated compounds, and synthesized ZnONPs. The previously reported procedure [44] was obeyed, using 100 µL of RBCs suspended in 10 mL of tris-buffered saline (TBS, pH 7.2). An initial concentration of the test sample solution at 4096 µg mL^−1^ was prepared in TBS. Subsequently, the serial dilution of twofold in a 96-well plate with a minimum concentration of 1 µg mL^−1^ was prepared. About 100 µL of the RBC suspension was reacted with equal amount of sample solutions (100 µL) and incubated with light shaking (37 °C, 30 min). 2% of triton x-100 and cell treated TBS were used as positive and negative control, respectively. The absorbance of released hemoglobin was noted at 540 nm after the centrifugation of supernatant of cell suspension for 10 min at 1500 rpm (Thermo Scientific Multiscan Spectrum). Blood agar was used to measure the MLC. A 10 µL × 2 of each of the dilutions that displayed no visible growth was loaded on a blood agar plate and incubated at 35 °C for 18 h. A decrease of 99.9% viable cells at minimum concentration was used to estimate the MLC. The percentage hemolysis was calculated using the stated equation:

Hemolysis rate (%) = (A − A_0_)/(A_100_ − A_0_) × 100%

where A, A_0_, and A_100_ denoted the sample, negative control, and positive control absorbances, respectively.

### 2.15. Anticancer Activity

An MTT assay method was employed to test the anticancer effect of ethyl acetate shoot fractional, catechin, curviflorside, curviflorin, and ZnONPs against the HeLa cancer cell line. The cancer cells were cultured in Dulbecco’s Modified Eagle Medium (DMEM) supplemented with 1% antibiotic and 10% of fetal bovine serum (FBS) solution in a humidified incubator with 5% CO_2_ supply at 37 °C. An MTT assay was used to determine cell inhibition. The HeLa cancer cells at1 × 10^5^ density were loaded into 96 well plates and incubated 37 °C for one complete day. Over the period of incubation, six concentrations (5, 10, 20, 40, 80, and 160 μg mL^−1^) of test samples were used to treat the cells for another 24 h at 37 °C. Then, 10 μL of MTT reagent solution was poured to each well and further incubated for 4 h at 37 °C. The formazan crystals (purple-colored) were acquired which were dissolved by the addition of dimethyl sulfoxide (DMSO, 100 μL). A multi-well ELISA plate reader was applied to measure the UV-Vis absorbance of the tested samples at 570 nm. The cancer cell inhibition percentage was acquired by the as-under stated equation:

Cell inhibition (%) = (100 − [A]_test_)/[A]_Control_ × 100

## 3. Results and Discussion

The formation of ZnONPs by the combination of herbal extract with a zinc salt solution, represents a congenial approach to the green synthesis of ZnONPs [45]. Antioxidants in herbal extracts, including polysaccharides, polyphenolics, flavonoids, terpenoids, tannins, alkaloids, saponins, vitamins, and amino acids, are reductive. Hence, herbal extracts (plants, fungi, bacteria, and marine sponges) serve as reducing, stabilizing and capping, agents that interact with the zinc salt solution to generate ZnONPs [46,47]. A study performed by Matinise et al. reported the preparation of ZnONPs using *Moringa oleifera* extract and the mechanism of formation. It was noticed that Zn^2+^ ions were generated due to the dissociation of Zn(NO_3_)2ž6H_2_O, and the L-ascorbic acid present in the *Moringa oleifera* extract was oxidized to L-dehydro ascorbic acid by free radicals [48]. Osuntokun et al. reported the mechanism of ZnONP formation using *Brassica oleracea* extract and revealed that hydroxyl group functionalities of the phenolic moiety of quercetin in extract reacted with Zn^2+^ in ZnCl_2_ solution to generate Zn(OH)_2_ [49]. Karnan and Selvakumar reported that the polyphenolic ellagic acid present in *Nephelium lappaceum* L. extract resulted in the formation of a stable zinc-elegant complex with Zn^2+^ (pH 5–7) that might be utilized for the preparation of ZnONPs by calcination at 450 °C [50]. It was established that aromatic hydroxyl functional groups could participate in complex stability with metal ions and resulted in the formation of metal oxide nanoparticles after calcination [51].

Herein, the ethyl acetate and methanolic extracts of *P. curviflorus* shoots were used to isolate pure constituents and the preparation of ZnONPs, respectively. Three triterpenes, namely catechin, curviflorside, and curviflorin, were isolated and identified using various chromatographic and spectroscopic techniques. The ZnONPs were biosynthesized using the methanolic biomass of *P. curviflorus* shoots and zinc acetate dihydrate with vigorous shaking for 20 min at ambient temperature. The precipitated ZnONP was centrifuged, repeatedly washed with methanol followed by de-ionized water, and oven-dried at 80 °C for 4 h. The prepared ZnONPs were characterized by various spectroscopic and microscopic techniques. A fluorescence technique based on ZnONPs with specific emission properties and desirable size was used for the detection of pure natural compounds (catechin, curviflorside, and curviflorin) isolated from *P. curviflorus* shoots extract.

### 3.1. Spectroscopic Characterization of ZnONPs

**Figure 2:**
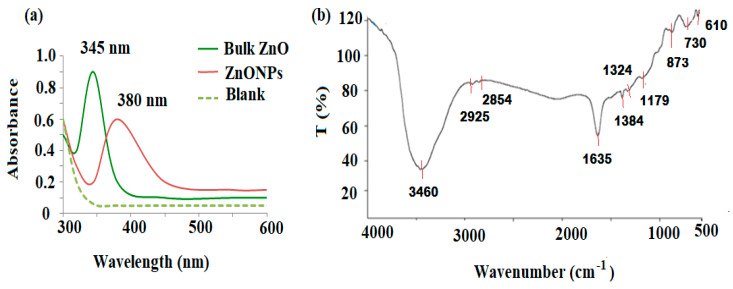
(**a**) UV-Vis, the absorption spectra at 345 and 380 nm for the bulk ZnO and ZnONPs, respectively; (**b**) FT-IR of ZnONPs synthesized using *P. curviflorus* biomass.

The UV spectral characteristics of the synthesized ZnONPs were investigated at scan wavelengths ranging from 300 to 600 nm, with respect to deionized water as blank. The formation of ZnONPs was confirmed by the strong blue shift absorption maximum at 345 nm with respect to the ZnO bulk of an absorption peak at 380 nm, indicating the decrease in the particle size of ZnO (Figure 2a). The FT-IR spectrum was evaluated at a resolution of 4000–500 cm^−1^ in the percent transmittance mode. The FT-IR spectrum of the synthesized ZnONPs displayed a fundamental mode of vibrations at 3460 cm^−1^ due to O-H stretching assigned to water absorption on the surface of zinc metal. Two vibration bands at 2925 and 2854 cm^−1^, which represents the C-H stretching vibrations, were also recognized in the spectrum. The vibration bands corresponding to symmetric stretching C=O, asymmetric C=O, and stretching C=O were recorded at 1635, 1384, and 1324 cm^−1^, respectively. The bond at 1179 cm^−1^ denoted the C-O stretching vibration. The formation of the tetrahedral coordination zinc atom was detected at 873 cm^−1^. The obtained peaks at 730 to 610 cm^−1^ indicate the stretching vibrations of ZnONPs (Figure 2b).

**Figure 3:**
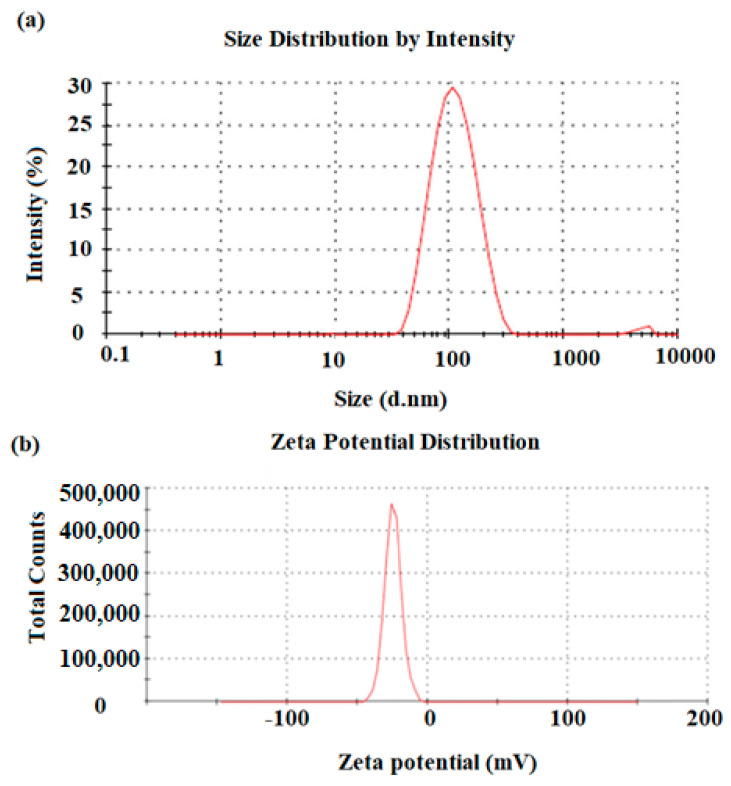
(**a**) DLS size distribution by intensity and (**b**) zeta potential distribution by mV of the ZnONPs pre-synthesized by *P. curviflorus* biomass.

A DLS method was used to determine the distribution and average particle size of the pre-synthesized ZnONPs. The particle size of the ZnONPs obtained was around 100 nm with a tetrahedral crystalline structure (Figure 3a). The surface charge and constancy of the green-synthesized ZnONPs were determined by zeta potential analysis. The results revealed that negatively charged groups present on the surface of ZnONPs were capping molecules that were involved in the average stability of the prepared nanoparticles. The estimation of Zeta potential depends on the movement of nanoparticles under the influence of the applied electric fields. The estimated average zeta potential value obtained for the ZnONPs was −28.4 mV, suggesting the high stability of ZnONPs (Figure 3b).

**Figure 4:**
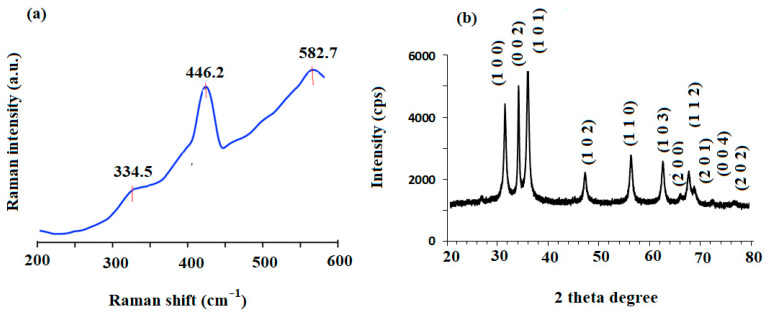
(**a**) Raman shift spectrum and (**b**) XRD pattern of ZnONPs synthesized using *P. curviflorus* biomass.

The measurement of Raman scattering was used to evaluate the vibrational characteristics of the ZnO nanostructures at room temperature. This is a non-destructive and very sensitive technique for examining semiconductor nanostructures. The materials containing ZnO wurtzite crystal structures mostly possess hexagonal systems with C6v4 (P63mc) and primitive cells with two formula units where all atoms inhabit C3v sites [52]. Raman spectroscopy exists in active and inactive photon modes, as claimed by the theory. In ZnO, the A1, E1, and 2E2 types represent the active photon mode, whereas the B1 type represents the inactive photon mode [53]. Figure 4a displays the Raman spectrum of biosynthesized ZnONPs. The observed peaks at 446.2 cm^−1^ and 334.5 cm^−1^ are due to the E2 (high) mode and 2E2 (medium) modes of wurtzite ZnO, respectively [53,54]. However, the appearance of a small peak at 582.7 cm^−1^ is a disorder related to the A1 longitudinal optical (A1 LO) mode, that was observed because of oxygen vacancies, structural defects, and Zn interstitials in ZnO [55].

Figure 4b demonstrates the X-ray diffraction pattern of the green-synthesized ZnONPs prepared from *P. curviflorus* biomass. The displayed results were measured using Cu Kα radiation (1.5406 Å) and collected at 2θ ranging from 20° to 80°. Significant peaks were recognized at angles θ of 31.73° (1 0 0), 34.36° (0 0 2), 36.19° (1 0 1), 47.49° (1 0 2), 56.57° (1 1 0), 62.76° (1 0 3), 66.38° (2 0 0) 67.96° (1 1 2), 69.04° (2 0 1), 72.64° (0 0 4), and 76.98° (2 0 2) planes which were revealed the presence of ZnO in nanosize. The sharp and narrow peaks in the biosynthesized ZnONPs confirm their crystalline structure. All diffraction peaks were in agreement with JCPDS Card No. 00-36-1451 [56]. Additionally, no significant peaks were noticed other than ZnO peaks indicating that the synthesized ZnONPs were free from impurities. The Scherrer equation, D = 0.9λ/(βCosθ), was applied for the calculation of mean crystallite size of the formed particles, where D is the mean size, λ is the wavelength (Cu Kα), β is the line broadening at half-maximum intensity (FWHM) of the ZnO (1 0 1) line, and θ is the Bragg angle. The average crystallite size of the biosynthesized ZnONPs was calculated to be 18.55 nm.

### 3.2. Microscopic Characterization

**Figure 5:**
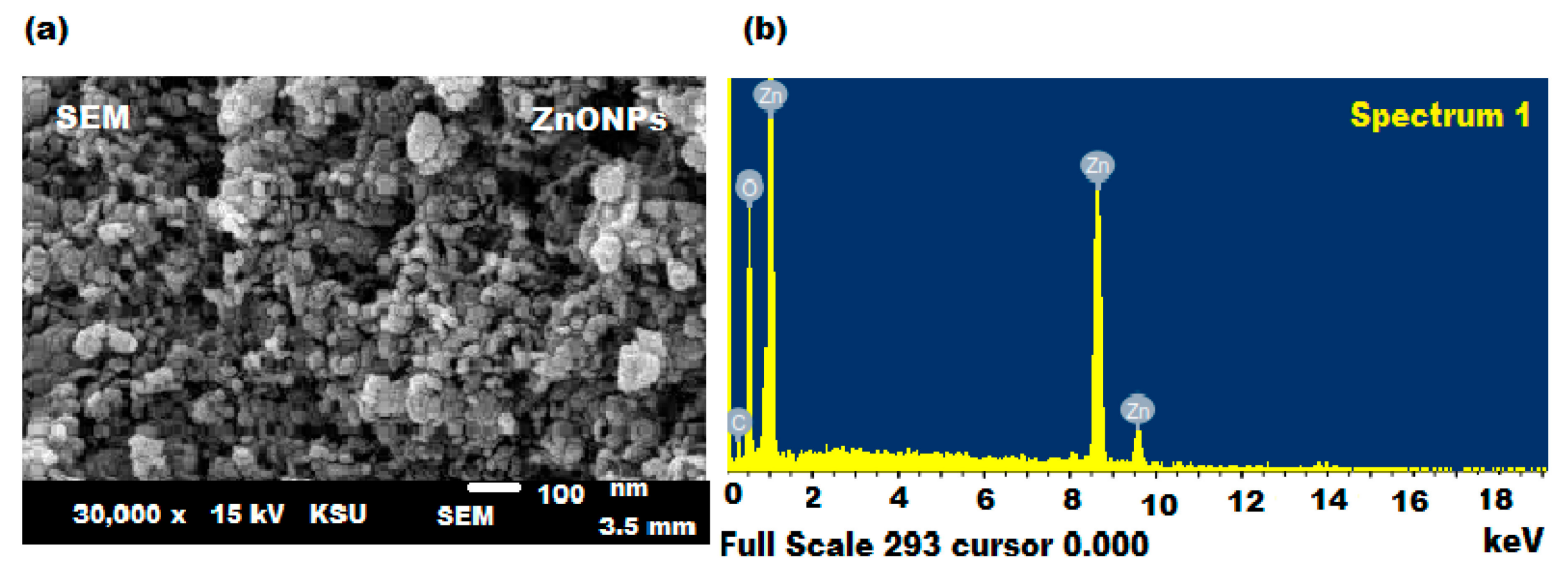
(**a**) SEM and (**b**) EDX spectra of biosynthesized ZnONPs.

SEM estimation was applied to study the morphological shape of the as-prepared ZnONPs and the selected images are shown in Figure 5a. It was observed that most of the pre-formed ZnONPs were hexagonal in shape and nanometer scale with an average diameter of around 80–100 nm. Moreover, it was noticed that all the prepared amounts of the ZnONPs were similar in dimension, together with an inadequate large particle. Additionally, a slight agglomeration was observed in the biosynthesized ZnONPs, which is predictable for the green synthesis of nanoparticles [57]. Furthermore, the EDX spectral analysis was conducted to examine the topographies of ZnONPs. The results showed that the prepared ZnONPs have a pristine ZnO phase [58]. The EDX examination of ZnONPs showed that the sample contains zinc at peaks around 1, 8.9, and 9.6 keV, whereas a single oxygen peak appeared at ~0.6 keV (Figure 5b) [59]. The high peak intensities of Zn and O confirmed that the sample contains around 94.32 w% of Zn and 5.68 w% O.

**Figure 6:**
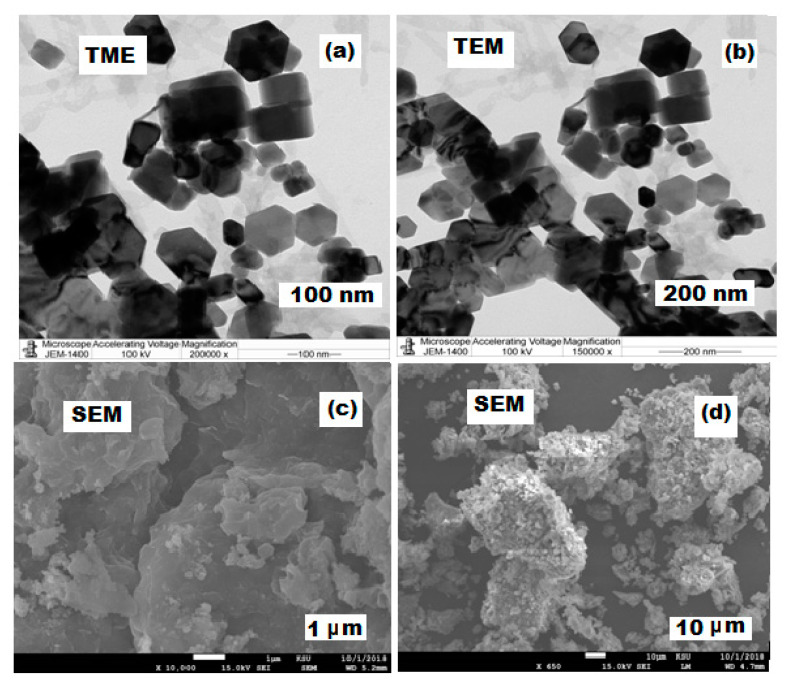
Microscopic images of ZnONPs at different magnifications. (**a**,**b**) The TEM images of ZnONPs at ×200,000 and ×150,000, 100 kV voltage. (**c**,**d**) SEM images at ×10,000 and ×500, 15.0 kV voltage.

Furthermore, different TEM and SEM magnifications were employed to study the morphological features and particle distribution of the prepared ZnONPs at ×200,000 and ×150,000, 100 kV voltage, and ×10,000, ×500, 15.0 kV voltage, respectively. It was noticed that the synthesized ZnONPs were hexagonal in shape, fairly distributed, and the results of TEM measurements provided a consistent size (79.8–80 nm) of pre-synthesized ZnONPs (Figure 6a,b). Moreover, Figure 6c,d showed the SEM images of the surface morphology of the formed nanoparticles.

### 3.3. Spectral Characteristics of Isolated Compounds

**Figure 7:**
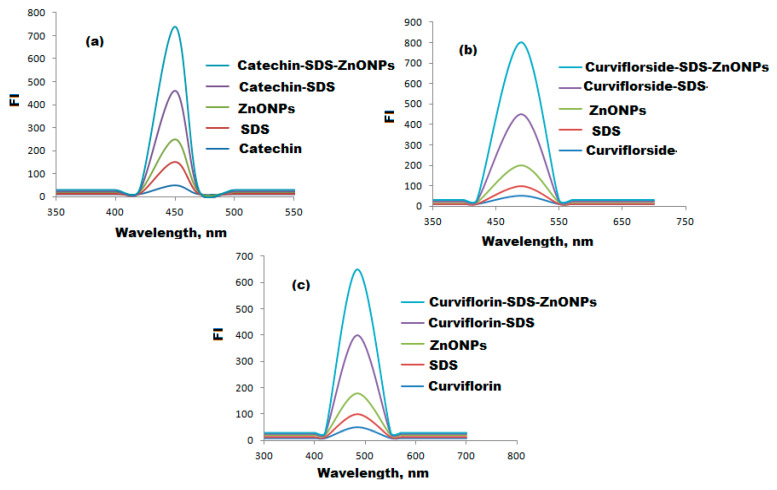
FI spectral characterization of (**a**) *catechin*-SDS-ZnONPs, (**b**) *curviflorside*-SDS-ZnONPs, and (**c**) *curviflorin*-SDS-ZnONPs systems in the presence of SDS and ZnONPs at λ_ex_ 380, 420, and 410 and λ_em_ 470, 490, and 484 nm.

All isolated compounds, catechin, curviflorside, and curviflorin displayed weak emission band strength in phosphate buffer at λ_em_ 470, 490, and 484 nm after excitation at λ_ex_ 380, 420, and 410, respectively. The emission band of each FL system was enhanced by the addition of ZnONPs (Figure 7). The FL analysis was applied to estimate the investigated compounds in the presenceof ZnONPs and catalyst sodium dodecyl suplate (SDS) and ZnONPs. The transfer of intramolecular energy from SDS to the ligand is an established phenomenon [60]. As demonstrated in Figure 7, high emission signals were displayed in the presence of SDS ions due to catechin, curviflorside, and curviflorin, respectively. The addition of colloidal ZnONPs to the systems under investigation has greatly increased FI. Moreover, the literature claims that the free space absorption condition of fluorophores can be modified by metal nanoparticles [61].

### 3.4. Selection of Optimum Experimental Conditions

**Figure 8:**
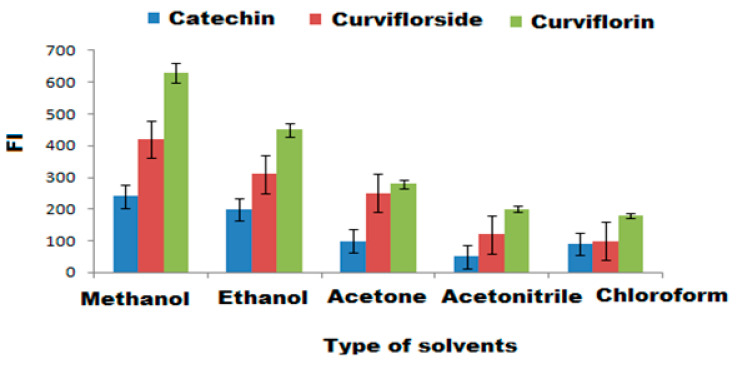
Selection of suitable diluting solvents using different kinds of organic solvents: methanol, ethanol, acetone, acetonitrile, and chloroform.

**Figure 9:**
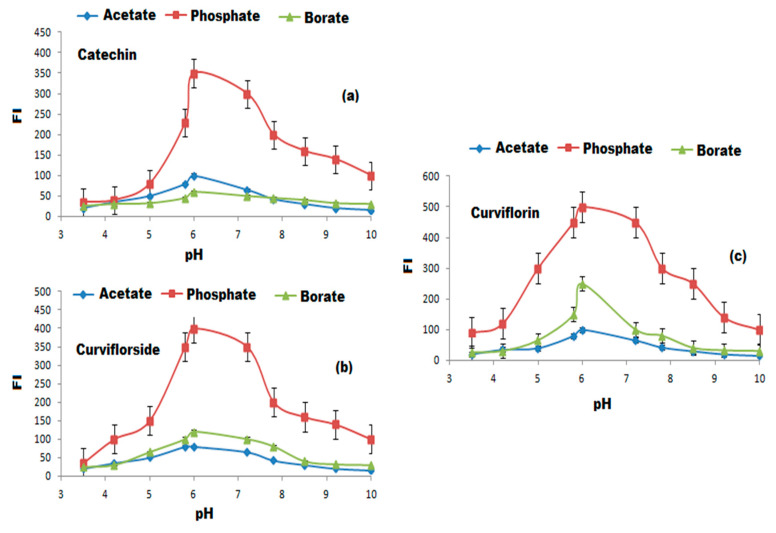
The selection of suitable buffers using 0.2 mol L^−1^ each of acetate, phosphate, and borate buffers on FI of (**a**) catechin, (**b**) curviflorside and (**c**) curviflorin.

**Figure 10:**
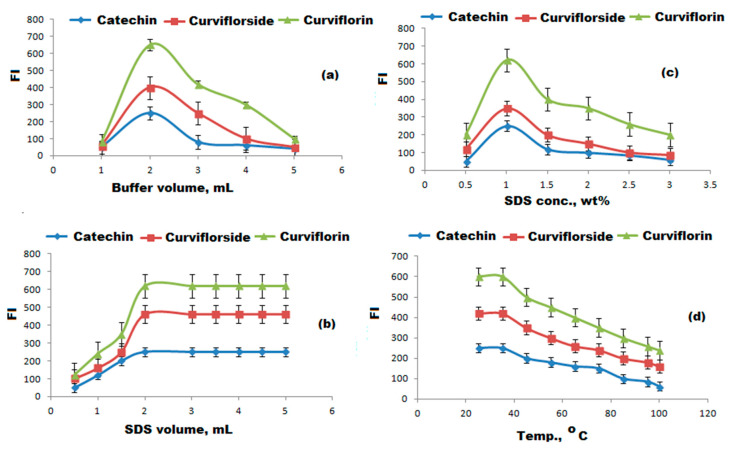
Optimization of FL conditions. (**a**) Effect of volume of buffer; (**b**) effect of volume SDS; (**c**) effect of SDS concentration; and (**d**) effect of temperature.

To select a suitable and eco-friendly solvent, different diluting solvents such as methanol, ethanol, acetone, acetonitrile, and chloroform were tested. The highest FI was achieved by diluting the test-isolated compounds with 3 mL of methanol (Figure 8). The influence of buffers the on the FI was tested over the pH range of 3–10 using 0.2 mil L−1 concentration towards borate, acetate, and phosphate buffers.The maximum FI was recorded after adding 0.2 mol L^−1^ of phosphate buffer of pH = 6 to the selected compounds (Figure 9a–c). The gradual increase in phosphate buffer volume (1.0–5.0 mL) was executed to examine the influence of buffer volume. The maximum FI was achieved by the addition of 2.0 mL of 0.2 mol L^−1^ phosphate buffer of pH 6 for the three suggested FL systems in the presence of SDS and ZnONPs. The effect of SDS concentration and volume on the FI systems in the presence of ZnONPs was tested in the range of 0.5–3% and 0.5–5.0 mL, respectively. The suitable volume and concentration of SDS to achieve the highest FI was 2.0 mL of 1.0% of SDS in the presence of 1.0 mL ZnONPs (Figure 10a–c). The effect of reaction time on the FI was investigated and the reaction was found to be dramatically faster, the FI attained a persistent value within 2 min, and stayed constant for 3 h for the three systems. On the other hand, the FI of the studied systems might be affected by increasing the temperature. The temperature effect was analyzed thermostatically in controlled temperature ( 25 to 100 °C range) on controlled water bath. The increase in temperature causes a decrease in the FI. Therefore, the room temperature was selected to carry out all the experiments (Figure 10d).

### 3.5. Analysis of Isolated Catechin, Curviflorside, and Curviflorin in Real Extract

The suggested FL method was successfully employed to determine catechin, curviflorside, and curviflorin in the parent extract. The suggested method displayed a linear concentration range of 10–120, 5–100, and 10–150 μg mL^−1^ with regression equations, I_FL_ = 3.6248 x + 20.47, I_FL_ = 4.9611 x + 10.527, and I_FL_ = 3.0359 x + 25.694 and correlation coefficients r = 0.998, 0.999, and 0.999 for the three natural isolated catechin, curviflorside, and curviflorin, respectively. The obtained results for catechin, curviflorside, and curviflorin were 40.8, 65.2, and 115.0 μg mL^−1^, respectively, with total yields of 16.3%, 26.1%, and 46% for the three isolated compounds in the *P.curiflorus* extract, respectively.

### 3.6. Antibacterial Activity

**Scheme 2:**
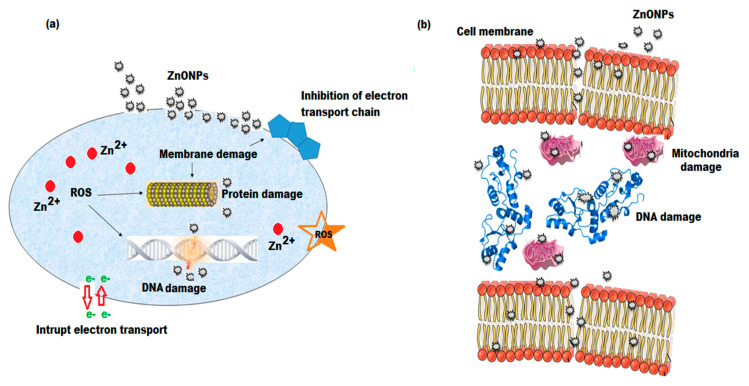
Schematic diagram of (**a**) antimicrobial and (**b**) anticancer mechanisms of ZnONPs.

**Figure 11:**
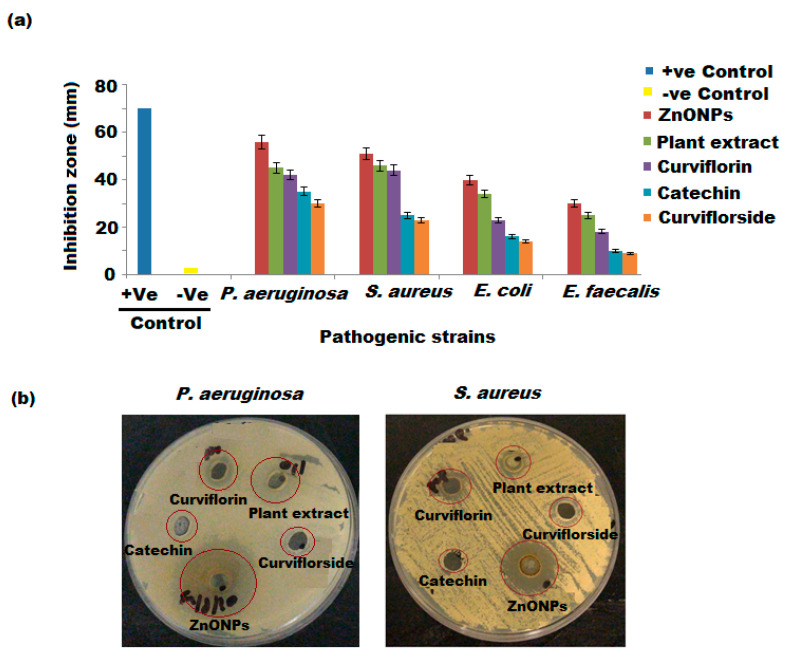
(**a**) Antibacterial activity of *P. curviflorus* extract, curviflorin, curviflorside, catechin, and ZnONPs against four pathogens. (**b**) ZOI against *P. aeruginosa* and *S. aureus* bacterial strains.

The antibacterial effects of biosynthesized ZnONPs, *P. curiflorus* extract, catechin, curviflorside, and curviflorin were examined against four different bacterial strains, including *E. faecalis*, *E. coli*, and *P. aeruginosa* at three different (30, 60, and 120 µg mL^−1^) concentrations. The average antibacterial potential of the investigated samples against pathogenic bacteria ranged from 15 mm to 30 mm. The interpretation of the results was based standard deviation of zone of inhibition mean diameter, Table 1. The results showed that the tested samples exerted antibacterial properties towards all four bacterial strains with the highest antibacterial effects against the *S. aureus* and *P. aeruginosa* strains (Figure 11a,b). The antibacterial activity of *P. curiflorus* extract against *S. aureus* and *P. aeruginosa* strains overlapped or were very much similar to pure curviflorin, with MIC values of 512 and 510 µg mL^−1^ (Table 2). The results are indicative of high content of curviflorin in *P. Curiflorus* extract, which appeared to be the potential antibacterial component isolated from the extract of *P. curiflorus*. On the other hand, the catechin and curviflorside showed a similar trend of antibacterial activity against all tested bacterial strains, with the greatest activity against *P. aeruginosa,* which is around four-fold lower than curviflorin and our tested *P. curiflorus* extract. However, the ZnONPs synthesized from *P. curiflorus* extract were the most effective against *S. aureus* and *P. aeruginosa*, with remarkable MIC values of 250 and 230 µg mL^−1^, respectively (Table 2). Several reports addressed in the literature have shown the promising antimicrobial potential of ZnONPs towards various Gram-positive and Gram-negative bacteria as well as spores [62,63]. The mechanism of the bactericidal effect of ZnONPs is still unclear. Numerous possible mechanisms have been suggested and featured, such as: (i) one of the convincible mechanisms depends on the grating ZnO surface, the electrostatic force between the ZnONPs, and bacterial surface straightforwardly eradicating the pathogens [64]; (ii) the ZnONP can interact with the lipid layer and cause cell membrane pulverization, which results in the loss of membrane uprightness and releases the cell content, finally leading to the death of bacterial cells; (iii) the release of Zn^2+^ ions from the ZnO structures; and (iv) the production of most reactive free radical species, including ROH, O_2_^2−^, H_2_O_2_, and OH^-^, that harm the DNA, cell proteins, and cell films, which may restrict bacterial development and consequently lead to the demise of bacteria [65]. The antibacterial properties of ZnONPs are influenced by the various dimensions of the particles, and smaller the particle size, higher is the antibacterial efficacy [66]. Other scientific studies have shown that microbial damage is caused by the disruption of the plasma membrane or by respiration blocking due to their interaction with oxygen and sulfhydryl group (SH) on the cell wall, leading to the exhaustion of intercellular ATP and cell death [67]. Thus, the ZnO nanoparticles are bio-safe, biocompatible, and non-toxic. The enhanced anti-*S. aureus* and *P. aeruginosa* effect of ZnONPs could be allocated to the large surface area of bioactive ZnONPs and the synergistic effect between the ZnONPs and isolated curviflorin. The possible mechanism of ZnO nanoparticles involves the enhancement of ROS production, increasing the peroxidation of the lipid membrane, resulting in membrane leakage reducing cell viability (Scheme 2a).

### 3.7. Morphological Study of P. aeruginosa and S. aureus (SEM)

**Figure 12:**
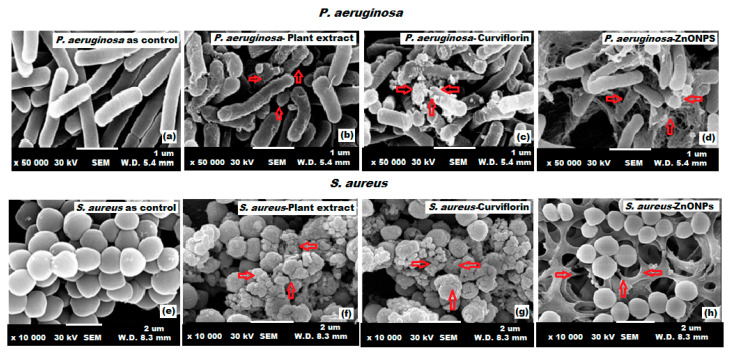
SEM images of *P*. *aeruginosa and S. aureus* treated with *P. curviflorus* extract (**b**,**f**), curviflorin (**c**,**g**), and ZnONPs (**d**,**h**) vs. untreated (**a**) *P*. *aeruginosa* (**e**) *S. aureus* strains.

The effect of *P. curiflorus* extract, curviflorin, and ZnONPs on the surface morphology of *P. aeruginosa* and *S. aureus* was investigated under SEM. The shape and size of the selected bacteria were changed upon treatment with *P. curiflorus* extract, curviflorin, and ZnONPs, as depicted in Figure 12. The ZnONPs showed the highest damage to bacterial cells, as they can easily penetrate the peptidoglycan membrane of *P. aeruginosa* and *S. aureus* due to their small size, causing the burst of the cell membrane, releasing the cell content, and subsequently cell demise occurs (Figure 12d,h) [68]. Untreated bacterial cells were chosen for comparison (Figure 12a,e).

### 3.8. Anti-Hemolytic Effects

Erythrocyte hemolysis is the disturbance of the membrane integrity of erythrocytes which causes the release of hemoglobin into the blood plasma surroundings. Erythrocytes are very sensitive to oxidative disturbances because of high concentration of hemoglobin and oxygen and the poly-saturated fatty acid content of the membrane that is supportive of oxidative processes [69]. These free radicals can easily form lipid peroxyl radicals, which attack the lipid layer of the cell membrane and convert them into hydroperoxides, causing adverse effects on the structure and function of the cell membrane [70]. The hemolytic activity of ZnO nanoparticles, *P. curiflorus* extract, and pure isolated compounds (catechin, curviflorside, and curviflorin) against human erythrocytes (RBCs) was measured to assess the toxicity of these test samples towards mammalian cells (Table 3). The ZnONPs showed the highest hemolytic activity, followed by *P. curiflorus* extract, whereas catechin and curiflorin displayed moderate hemolytic activity at a similar concentration of 1500 μg mL^−1^. However, curviflorside did not exert any hemolytic effect, even at the highest tested concentration of 8192 μg mL^−1^. The selectivity was defined as a ratio of hemolytic activity (HC_50_) to MIC and was calculated to access the bacterial cell selectivity of *P. curiflorus* compounds, ZnONPs, and *P. curiflorus* extract. As shown in Table 2, the hemolytic activity of ZnONPs and extract was less selective and curviflorside was most selective against all four bacterial strains. Whereas the selectivity of catechin and curviflorin varied between bacterial strains, with catechin showing equal levels of selectivity against *E. faecalis*, *E. coli,* and *P. aeruginosa*, and curviflorin against *S. aureus* and *P. aeruginosa* strains. The oxidative damage caused by ZnONPs in the erythrocytes can be attributed to their small size and the presence of Zn2+ ions surrounded by several –OH groups in phenolic compounds, or the fact that the –NH2 or Ar-OH in amines present in the plant extract displayed a strong antioxidant effect which prevented oxidation by free radicals [71].

### 3.9. Anticancer Activity

**Figure 13:**
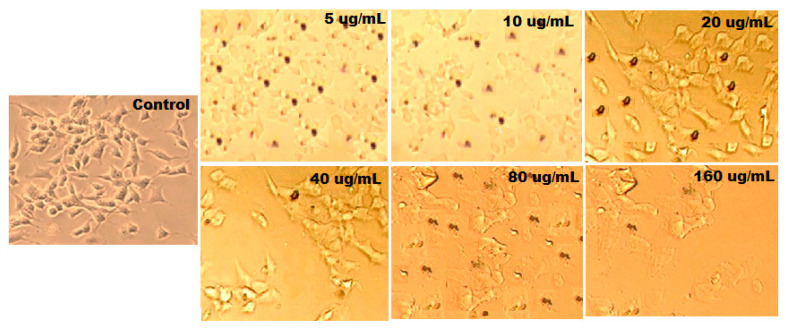
Microscopic images of HeLa cells treated with ZnONPs at different concentrations (5, 10, 20, 40, 80, and 160 μg) for 48 h.

The anticancer potentials of ethyl acetate shoot fraction, catechin, curviflorside, curviflorin, and ZnONP were evaluated against the HeLa cancer cell line. The results obtained showed that the test samples expressed anticancer potential in a dose-dependent manner. The cell inhibitions of ethyl acetate shoot fraction, catechin, curviflorside, curviflorin, and ZnONPs at different concentrations (5, 10, 20, 40, 80, and 160 μg mL^−1^) were observed under an optical microscope and are presented in Figure 13. The results showed that the ZnONPs displayed excellent anticancer effects, followed by curviflorin and the ethyl acetate shoot fraction. However, catechin and curviflorside exerted moderate effects. The ZnONPs produce reactive oxygen species and increase the inhibition of cervical tumor cells. A 160 μg mL^−1^ concentration of ZnONPs induces 64% cancer cell demise. The anticancer potential of ZnONPs might involve two possible mechanisms and contribute to the disruption of the HeLa cancer cell membrane [72,73]. Durin this reaction process, the biosynthesized nanomaterials attach to the negatively charged tumor cell membrane. Further, they release positively charged Zn^2+^ ions that cause cell wall disruption. The charged surface of the nanomaterials plays a crucial role in this mechanism. Another mechanism involved in the anticancer effect of the nanoparticles is the production of free radical oxygen species (-OH, O_2_^2−^, H_2_O_2_, etc). These ROS interact with the negatively charged cell membrane, leading to cell demise. Consequently, the anticancer effect of the nanomaterials depends on the distribution and shape of the nanoparticles that peretrate into thecancerous cell. The small-sized nanoparticles can easily penetrate into the cancer cells, leading to the damage of DNA and mitochondrial functions and finally inducing the death of the cancer cells (Scheme 2b). Additionally, the nanoparticles authorize ROS and oxidative stress to play an important role in the inhibition of cancer cells [74]. The mechanism behind the anticancer potential of ZnONPs is revealed in Scheme 2b. Thus, the obtained results show that the ZnONPs exhibited higher inhibition of the growth of cancer cells. Furthermore, the biogenic synthesis of ZnONPs displayed exceptional anticancer effects in contrast to plant extracts and pure isolated constituents (catechin, curviflorside, and curviflorin). The anticancer effect of ZnONPs tested with HeLa cancer cells at different concentrations is presented in Table 4.

## 4. Conclusions

In conclusion, we have developed ultrasensitive fluorescent ZnONPs with enhanced fluorescence using *P. curviflorus* extract. The ZnONPs showed high sensitivity, selectivity, low cost, and efficiency in sensing the three biomarkers (catechin, curviflorside, and curviflorin) isolated from the shoots of *P. curviflorus*. The developed analytical method is a maiden method with excellent sensitivity and reliability of the concurrent analysis of isolated bioactive markers catechin, curviflorside, and curviflorin in *P. curviflorus* extract. The antibacterial, anti-hemolytic, and anti-cancer properties of the extracted isolated compounds (catechin, curviflorside, and curviflorin) and the green-synthesized ZnONPs were studied and the obtained results showed that all the tested samples possess significant antibacterial and anticancer properties against *S. aureus*, *E. faecalis*, *E. coli*, and *P. aeruginosa* bacterial strains and HeLa cancer cells, respectively. Among all the investigated samples, the green-synthesized ZnONPs exhibited the highest antibacterial and anticancer activities. Hence, ZnONPs can be effectively used for detecting catechin, curviflorside, and curviflorin as well as a future replacement for antibiotics.

## Figures and Tables

**Table 1:** Antibacterial activity of *P.* *curiflorus* extract, catechin, curviflorside, curviflorin, and ZnONPs against four pathogenic strains.

Samples	Concentration (µg mL^−1^)	Zone of Inhibition (ZOI)			
		*S. aureus*	*E. faecalis*	*E. coli*	*P. aeruginosa*
*P. curiflorus* extract	120	18.7 ± 0.5	9.2 ± 0.8	15.0 ± 1.2	20.6 ± 0.5
Catechin	120	14.2 ± 0.6	11.3 ± 0.5	14.2 ± 1.1	16.9 ± 0.5
Curviflorside	120	16.3 ± 0.5	10.2 ± 0.8	13.5 ± 0.4	18.6 ± 0.7
Curviflorin	120	19.2 ± 0.5	9.8 ± 1.2	15.4 ± 0.8	21.2 ± 0.9
ZnONPs	120	28.2 ± 1.1	15.1 ± 0.8	17.2 ± 0.52	30.1 ± 0.48
Positive control (Ciprofloxacin)	120	32.6 ± 2.1	17.9 ± 1.5	21.3 ± 1.2	33.4 ± 1.4
Negative control (Dist. Water)	-	0.00	0.00	0.00	0.00

**Table 2:** MIC and MLC of the *P.* *curiflorus* extract, catechin, curviflorside, curviflorin, and ZnONPs.

Sample	*S. aureus*		*E. faecalis*		*E. coli*		*P. aeruginosa*	
	MIC (μg/mL)	MLC (μg/mL)	MIC (μg/mL)	MLC (μg/mL)	MIC (μg/mL)	MLC (μg/mL)	MIC (μg/mL)	MLC (μg/mL)
Plant extract	512	1024	1024	2048	1024	2048	510	1024
Catechin	2048	4096	1024	≥8192	1024	2048	1024	2048
Curvilforside	2048	4096	2048	≥8192	2048	4096	1024	2048
Curviflorin	512	1024	1024	4096	2048	2048	510	2048
ZnONPs	250	625	823	1525	815	1510	230	560
Gentamicin	8	10	8	10	5	8	5	5
Erythromycin	1	60	130	540	78	500	1	30
Negative control (Dist. Water)	0.00	0.00	0.00	0.00	0.00	0.00	0.00	0.00

**Table 3:** Hemolytic activity of *P.* *curiflorus* extract, catechin, curviflorside, curviflorin, and ZnONPs.

Sample	Hemolytic Activity HC_50_ (μg/mL)	Selectivity (HC_50_/MIC)			
		*S. aureus*	*E. faecalis*	*E. coli*	*P. aeruginosa*
Plant extract	52	0.8	0.6	0.3	0.1
Catechin	1500	0.7	1.5	1.5	1.5
Curviflorside	≥8192	≥4	≥4	≥4	≥8
Curviflorin	1500	3	1.5	0.7	3
ZnONPs	28	0.1	0.02	0.02	0.05

**Table 4:** The anticancer activity of ZnONPs tested with the HeLa cell line by the MTT assay method at different concentrations.

Concentration of ZnONPs (µg/mL)	Absorbance (nm)	% Inhibition of HeLa Cell Line
160	0.250	64.06
80	0.305	54.35
40	0.382	41.85
20	0.430	32.82
10	0.485	22.06
5	0.492	12.02
Control	160	0

## Data Availability

The data used to support the findings of this study are included within the article.
